# Risk factors associated with fatal influenza, Romania, October 2009 – May 2011

**DOI:** 10.1111/irv.12209

**Published:** 2013-11-20

**Authors:** Laurentiu Zolotusca, Pernille Jorgensen, Odette Popovici, Adriana Pistol, Florin Popovici, Marc-Alain Widdowson, Viorel Alexandrescu, Alina Ivanciuc, Po-Yung Cheng, Diane Gross, Caroline S Brown, Joshua A Mott

**Affiliations:** a Ministry of HealthBucharest, Romania; b WHO Regional Office for EuropeCopenhagen, Denmark; c National Institute of Public HealthBucharest, Romania; d Influenza Division, U.S. Centers for Disease Control and PreventionAtlanta, GA, USA; e Cantacuzino Institute on Research and Development on Microbiology and ImmunologyBucharest, Romania

**Keywords:** Influenza, risk factors, Romania, severe acute respiratory illness, surveillance

## Abstract

**Background:**

Limited data are available from Central and Eastern Europe on risk factors for severe complications of influenza. Such data are essential to prioritize prevention and treatment resources and to adapt influenza vaccination recommendations.

**Objectives:**

To use sentinel surveillance data to identify risk factors for fatal outcomes among hospitalized patients with severe acute respiratory infections (SARI) and among hospitalized patients with laboratory-confirmed influenza.

**Methods:**

Retrospective analysis of case-based surveillance data collected from sentinel hospitals in Romania during the 2009/2010 and 2010/2011 winter influenza seasons was performed to evaluate risk factors for fatal outcomes using multivariate logistic regression.

**Results:**

During 2009/2010 and 2010/2011, sentinel hospitals reported 661 SARI patients of which 230 (35%) tested positive for influenza. In the multivariate analyses, infection with influenza A(H1N1)pdm09 was the strongest risk factor for death among hospitalized SARI patients (OR: 6·6; 95% CI: 3·3–13·1). Among patients positive for influenza A(H1N1)pdm09 virus infection (*n* = 148), being pregnant (OR: 7·1; 95% CI: 1·6–31·2), clinically obese (OR: 2·9;95% CI: 1·6–31·2), and having an immunocompromising condition (OR: 3·7;95% CI: 1·1–13·4) were significantly associated with fatal outcomes.

**Conclusion:**

These findings are consistent with several other investigations of risk factors associated with influenza A(H1N1)pdm09 virus infections. They also support the more recent 2012 recommendations by the WHO Strategic Advisory Group of Experts on Immunization (SAGE) that pregnant women are an important risk group for influenza vaccination. Ongoing sentinel surveillance can be useful tool to monitor risk factors for complications of influenza virus infections during each influenza season, and pandemics as well.

## Background

Limited data are available from Central and Eastern Europe on populations at risk for severe complications of influenza. Such data are needed to better inform the expansion of local influenza vaccine programs. In this report, we analyze the first 2 years of sentinel hospital-based severe acute respiratory illness (SARI) surveillance data in Romania, which has a locally manufactured vaccine on the market.^1^,^2^ We undertake a retrospective study of risk factors for fatal outcomes among all SARI patients and among those who tested positive for influenza.

## Methods

**Figure 1:**
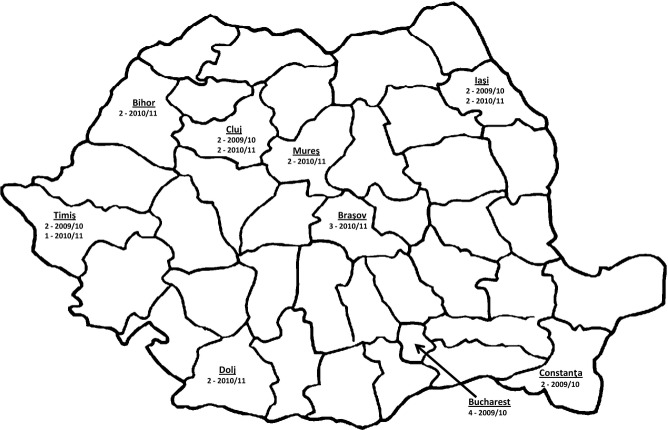
Number and location of SARI sentinel hospitals in Romania, by year of surveillance initiation, 2009/2010 and 2010/2011.

During October 2009, sentinel SARI surveillance was initiated in 14 infectious disease and pediatric wards of 12 county hospitals located in five of 42 counties in Romania. In October 2010, this surveillance system was expanded to include 26 hospitals and 38 wards in nine counties (Figure 1). Clinicians in the hospitals participated in surveillance on a voluntary basis. To implement the surveillance, each hospital assigned a hospital epidemiologist that worked three hours per day to oversee case definition adherence, data collection, respiratory specimen collection and transport, and reporting to the county health authority. The clinical case definition for SARI in persons ≥5 years of age was as follows: onset of orally measured fever >38°C, cough or sore throat, and shortness of breath or difficulty in breathing within 7 days prior to hospital admission. For children aged <5 years, the WHO Integrated Management of Childhood Illnesses clinical criteria for respiratory signs were applied.^3^

Epidemiological data considered in these analyses included patient date of birth (age); sex; week of hospitalization; time from onset to care-seeking; time from onset to specimen collection; signs and symptoms present on admission; pre-existing conditions (asthma, chronic pulmonary disease, cardiac disease, diabetes, immunocompromised status, hepatic disease, renal disease, and clinicians' judgement of clinical obesity); pregnancy status (reported by female patients of reproductive age); history of monovalent or trivalent influenza vaccination during the 2009/10 and 2010/11 seasons; oseltamivir use at time of case detection, and outcome (death or discharge).

Nasopharyngeal and oropharyngeal swab specimens were collected from detected SARI patients during weeks 40/2009–20/2010; and 40/2010–20/2011. Swabs were placed in a single viral transport media and sent to the Cantacuzino Institute on Research and Development 2 on Microbiology and Immunology in Bucharest, Romania for confirmatory diagnostic testing. The specimens were analyzed by rtRT-PCR for influenza A(H1N1)pdm09, seasonal influenza A(H1N1) and A(H3N2), and influenza B using the WHO CDC reagent kit for influenza diagnostics.^3^

We conducted bivariate and multivariate logistic regression analyses to evaluate risk factors for fatal outcomes in patients hospitalized with (i) SARI and (ii) SARI that tested positive for influenza virus infections. When evaluating risk factors for fatal outcomes due to SARI, we considered laboratory-confirmed infection with influenza A(H1N1)pdm09, A(H3N2), or influenza B as independent risk factors for fatal outcomes in comparison with a reference group of influenza-negative persons. All variables associated with a fatal outcome in the bivariate analyses at a significance level of *P* < 0·25 were included in the multivariate model. Using backward stepwise elimination, variables that were not significantly associated (i.e., *P* > 0·05) with a fatal outcome were then excluded. For all variables that were statistically significant, we evaluated their interaction with influenza infection independent of any main effects to predict a fatal outcome for SARI. All risk factor analyses were performed with Stata 10.0 (StataCorp, College Station, TX, USA).

## Results

**Figure 2:**
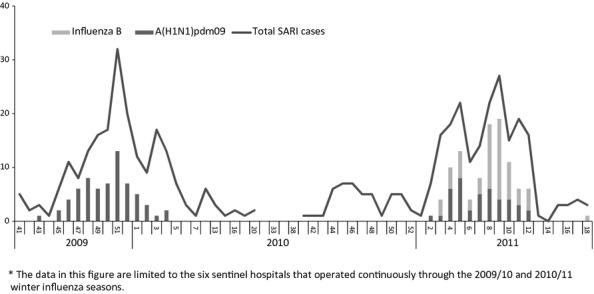
SARI admissions and influenza detections by week, Romania, 2009/2010 and 2010/2011 winter influenza seasons. The data in this figure are limited to the six sentinel hospitals that operated continuously through the 2009/10 and 2010/11 winter influenza seasons.

A total of 661 SARI patients were identified during the two seasons. Fifty-five percent (360/661) were male, the median age was 22 (range 0–86 years). Of 661 SARI cases, 230 (34·8%) tested positive for influenza. Of these 230 positive cases, 148 (64·3%) tested positive for A(H1N1)pdm09, 81 (35·2%) for influenza B, and 1 (0·4%) for A(H3N2) (Figure 2). Of the 148 influenza A(H1N1)pdm09 viruses, 19 (12·8%) were from SARI patients aged 0–4 years, 121 (81·8%) from patients 5–64 years, and eight (5·4%) from patients 65 years and older. The 81 influenza B patients (all from 2010/2011) included 20 (24·7%) aged 0-4, 54 (66·7%) aged 5–64, and seven (8·6%) aged 65 years and older. Of 648 SARI patients with information on influenza vaccination 11 (1·7%) reported receiving either the 2009 monovalent or the 2010/2011 trivalent influenza vaccines. The mean (median) time from symptom onset to NP/OP swab collection was 4 (4) days for both influenza-positive and influenza-negative SARI cases.

### Factors associated with fatal outcomes in patients hospitalized with SARI

Of 661 SARI cases, 44 (6·7%) were fatal. Thirty-two (72·7%) of the 44 fatal SARI cases were laboratory-confirmed to have influenza virus infections [29 influenza A(H1N1)pdm09 and three influenza B] indicating the overall case-fatality percentage (CFP) of 13·9% (32 deaths/230 influenza hospitalizations). The observed CFP was 19·6% (29/148) for influenza A(H1N1)pdm09 infections and 3·7% (3/81) for influenza B infections.

In the multivariate model, there were 659 SARI cases with complete data. The single influenza A(H3N2) case was excluded from analysis. Influenza A(H1N1)pdm09 infection was strongly and independently associated with a fatal outcome. Other variables significantly and independently associated with a fatal outcome were being pregnant, having hepatic disease, increasing age in years, and increasing time from onset to hospital admission. Influenza B was not significantly associated with fatal outcomes relative to other causes of SARI hospitalizations. None of the variables displayed significant interactions with influenza A(H1N1)pdm09 infection to predict fatal outcomes from SARI.

### Risk factors associated with fatal outcomes in SARI patients testing positive for influenza A(H1N1)pdm09

Analyses of risk factors for fatal outcomes among those testing positive for influenza were limited to patients testing positive for influenza A(H1N1)pdm09, as this was the only influenza type or subtype that included an analyzable sample size. In the multivariate model, pregnancy, clinical obesity, having an immunocompromising condition, and hepatic disease (*P* = 0.057) were independent risk factors for a fatal outcome among influenza-positive patients (Table 1).

**Table 1:** Multivariate analyses of risk factors associated with a fatal outcome among SARI patients that were laboratory-confirmed to have influenza A(H1N1)pdm09 virus infections (*n* = 148)

Variable (*n* = 148)	Percent with fatal outcome	OR	95% CI	*P*-value
Pregnancy				
No (*n* = 139)	18·0	1·00	1·62–31·17	0·01
Yes (*n* = 9)	44·4	7·10		
Immunocompromised				
No (*n* = 636)	6·1	1·00	1·05–13·38	0·042
Yes (*n* = 23)	21·7	3·74		
Hepatic disease				
No (*n* = 135)	17·8	1·00	0·97–12·19	0·057
Yes (*n* = 13)	38·5	3·43		
Clinically obese				
No (*n* = 112)	17·0	1·00	1·61–31·17	0·01
Yes (*n* = 36)	27·8	2·89		

## Discussion

In Romania during 2009–2011, influenza A(H1N1)pdm09 virus infection was a strong predictor of fatal outcomes in persons with hospitalized SARI. Patients reported to be clinically obese, pregnant women, and persons with immune-compromising conditions were more likely to die from influenza than others hospitalized with influenza. These findings are consistent with other investigations of risk factors associated with influenza A(H1N1)pdm09 infections^4^–^8^ and also support the 2012 recommendations by the WHO Strategic Advisory Group of Experts on Immunization that pregnant women are an important risk group for influenza vaccination.^9^ The percentage of SARI patients that received monovalent or trivalent influenza vaccine (1·7%) was lower than coverage rates observed in the Romanian population during the study period (2009/10 – 5·2 trivalent,^10^ 8·0% monovalent;^11^ 2010/11 – 5·6% trivalent^12^). Thus, it is also conceivable that expanded vaccine uptake in these population subgroups could further reduce influenza-associated severe outcomes in Romania.

The CFP of 19·6% for influenza A(H1N1)pdm09 infections was higher than CFP of 7% reported in the United States^13^ or 14·3% in Australia and New Zealand.^14^ While the CFP may have been higher in Romania, other factors may also account for this finding. Only public hospitals are represented in this surveillance system, and it is unclear if the CFP would remain similar if private facilities were included. Given the voluntary nature of the surveillance and limited work-hours of hospital epidemiologists, it is also possible that a more severe proportion of admitted SARI patients were detected by participating clinicians, and this could have varied by ward or patient type. However, as no rapid diagnostic tests were in use in the sentinel hospitals, doctors would have been unaware of the influenza status of SARI patients at the time of admission or case reporting.

Other possible limitations to these findings include the requirement of measured fever in the SARI case definition. This may have limited sensitivity to detect influenza in the old and young, who may be less likely to present with fever. PCR testing may also have missed persons, particularly elderly persons, presenting with sequelae of earlier influenza infections in whom viruses could no longer be detected. Our measurement of treatment, including oseltamivir use, occurred at time of case detection and was possibly not comprehensive. We also had limited statistical power to determine whether the risk factors for fatal influenza were different from those for fatal respiratory disease in general. We also cannot yet review differences in hospital-specific mortality or look at risk for death due to subtypes of influenza other than A(H1N1)pdm09. As testing for influenza only occurred during the periods of weeks 40–20, we may have missed influenza cases outside of this time period. Finally, we did not collect information on the ethnic status of patients, so we were unable to determine whether potentially vulnerable population subgroups (e.g., the Roma population) were at higher risk of fatal outcomes.

The quality of routine surveillance data from systems relying on over-stretched clinicians to detect and report cases may be limited in comparison with data collected in targeted research programs. However, the consistency of findings from these data with other published studies on risk factors for fatal influenza supports the use of ongoing surveillance data to monitor virological trends in the epidemiology of influenza and to serve as a tool to monitor risk factors for complications of influenza virus infections during each influenza season.
